# Associations between college/university campus characteristics and student body mass index

**DOI:** 10.1265/ehpm.21-00352

**Published:** 2022-03-19

**Authors:** Caitlin P. Bailey, Angelo F. Elmi, Mary T. Hoban, Christine Kukich, Melissa A. Napolitano

**Affiliations:** 1 Department of Prevention and Community Health, The George Washington University Milken Institute School of Public Health, Washington, DC, USA; 2 Department of Biostatistics and Bioinformatics, The George Washington University Milken Institute School of Public Health, Washington, DC; 3 American College Health Association, Silver Spring, Maryland, USA

**Keywords:** College/university, Student, Body mass index, US region

## Abstract

**Background:**

Campus environments are associated with undergraduate weight. However, few studies have examined campus type and geographic location in relation to student weight. This article aimed to identify college/university students with elevated BMIs by campus type and region.

**Methods:**

Linear mixed effects regression models were fit to data from the American College Health Association-National College Health Assessment II. Analyses tested associations between campus type/region and student self-reported BMI.

**Results:**

The sample included 404,987 students from 445 schools with mean BMI 24.9 ± 5.8. Across all school types/regions, BMI confidence intervals included overweight values. Two-year and public school students had higher BMIs compared to four-year and private school students, respectively. Students in the Midwest had higher BMIs compared to students in the Northeast. In the South only, Minority Serving Institution (MSI) students had higher BMIs compared to non-MSI students.

**Conclusion:**

Healthy weight maintenance programs should be made available to undergraduate students.

## Background

Since 1999, the prevalence of obesity (body mass index [BMI] ≥ 25) in the United States has steadily risen from 30% to just over 42% [1]. Obesity is associated with serious health consequences, including diabetes, heart disease, and thirteen cancers [2]. Furthermore, obesity and its sequelae are increasingly occurring among young adults (e.g., ages 20–39), indicating a need for obesity prevention efforts in this age group [3, 4].

In the US alone, close to 19.6 million young adults are enrolled in higher education [5]. The average undergraduate student gains an estimated 3.5–6.5 pounds during four years of study [6, 7]. Furthermore, the majority of college/university students do not meet dietary and physical activity guidelines [8]. College health literature indicates that campus environments may play a role in promoting student weight gain and unhealthy weight-related behaviors [9]. However, most studies exploring college student weight and/or weight-related behaviors in the context of college environments have been conducted in small, convenience samples of majority white, female, four-year students and do not incorporate data from multiple schools [9]. This leaves a gap in our understanding of how different school types (e.g., two-year, public, Minority-Serving Institution [MSI]) are associated with student health outcomes. For example, data on college/university student weight is lacking among two-year schools and MSI, which disproportionately serve public health priority populations [9].

Two-year school students in the US serve a greater proportion of minority students compared to four-year school students [10]. Two-year school students are also older, more often female, more often part-time, and work for pay more often than their four-year counterparts [10]. Similarly, students attending public schools are more likely to represent minority groups (i.e., Hispanic, Asian, American Indian/Alaskan Native, or two or more races) and come from families of lower socio-economic status compared to private school students [11, 12]. Fewer data are published on students attending MSI, in part due to inconsistent definitions of what constitutes MSI eligibility. However, in comparison to non-MSIs, MSIs do serve a greater proportion of students representing minority groups.

In addition to demographic differences, studies suggest weight and weight-related behaviors differ across school types. Students attending two-year schools have reported higher prevalence of overweight/obesity, lower physical activity levels, more television viewing, and more soda and fast-food consumption compared to four-year students [13, 14]. Subsequent studies of other two-year school populations corroborate the high prevalence of students at-risk of developing overweight/obesity [15–17]. There is also some evidence indicating that two-year school students experience greater rates of food insecurity than four-year school students, a circumstance associated with overweight status among women [18, 19]. Additionally, socioeconomic factors (e.g., parental education, financial stress) have been associated with poor dietary and physical activity behaviors of college students [17]. Finally, little is known about weight and weight-related behaviors and circumstances of college/university students attending MSI; however, a large proportion of MSIs are located in the US South and regional differences in overweight/obesity indicate that students attending college/university in the South may be at increased risk, regardless of school type [20, 21].

This study aims to identify student subgroups by campus type and region with elevated BMIs in need of public health surveillance and targeted interventions. Despite the demographic differences associated with school type and weight-related behaviors among college/university students, little is known about student weight status across multiple campus types nationally. Thus, this study contributes to an important gap in the literature regarding potential associations between campus type (i.e., two-year, public, MSI) and college student BMI. Our hypotheses are as follows: 1) students attending two-year schools will have greater BMIs than students attending four-year schools, 2) students attending public schools will have greater BMIs than students attending private schools, 3) students attending MSIs will have greater BMI than students attending non-MSIs, and 4) there will be regional differences in student BMI across school types.

## Methods

### Study design

Data from the American College Health Association-National College Health Assessment II (ACHA-NCHA II) survey (2015–2019) were used to test hypotheses. The ACHA-NCHA II is a serial cross-sectional dataset of health behaviors collected from college/university students clustered by academic institution. At the campus-level, the data represent a diverse, self-selected sample of colleges/universities from all 50 states and Washington, D.C. Within each college/university, students are either sampled randomly or via census [8]. Institutions provide self-report data on campus-level variables, such as campus type and US region. Students provide self-report responses to survey questions designed to measure a range of health-related factors. The ACHA-NCHA II data was made available upon request and contained no individually identifiable information; therefore, this project did not meet the definition of human subjects research and did not require further review by the institutional review board of the first author’s university.

### Statistical analysis

A linear mixed effects regression model was fit to data from the American College Health Association-National College Health Assessment II (ACHA-NCHA II; 2015–2019). Regression analyses were performed in RStudio using the “lme4” package [22]. Analyses tested associations between campus-level characteristics and student BMI, including interactions between campus type and US region, holding constant age, gender, race/ethnicity, hours worked per week, and school ID (deidentified). Contrast tests using Scheffé-adjusted probabilities for pairwise comparisons were used to test and visualize interactions between campus-level characteristics and US region. Backward selection was used to find the most parsimonious set of interactions.

*Outcome.* The outcome variable, student BMI, was calculated from students’ self-reported height and weight (kg/m^2^). The outcome was log transformed to fit the data, as determined by QQ plots.

*Predictors.* Explanatory variables were three binary variables, each indicating a campus-level characteristic of interest: 1) two-year vs. four-year status, 2) public vs. private status, 3) MSI vs. non-MSI status. A four-level categorical predictor, US region (Northeast [NE], Midwest [MW], South [S], West [W]), was entered as an interaction term with each campus-level characteristic. Predictor variables were self-reported by campus administrators and verified by ACHA. In this study, MSIs are defined using the ACHA definition (i.e., institutions with one or more of the following designations: HBCUs, Predominantly Black Institutions, Hispanic Serving Institutions, Tribal Colleges and Universities, Native American Non-Tribal Institutions, Alaskan Native- or Hawaiian-Service Institutions, Asian American- and Native American Pacific Islander-Serving Institutions, or Institutions with High Hispanic Enrollment).

*Covariates.* Age, gender, race/ethnicity, and hours worked per week were entered into the model as fixed effects covariates. School ID, a random, deidentified, unique number for each campus, was entered as a random effect covariate to account for potential differences by campus.

## Results

The analytic sample included n = 404,987 students from 445 unique schools. The mean student BMI was 24.9 (SD 5.8). Students were majority female (67%), non-Hispanic White (61%), and attended public schools (66%). Thirty-nine percent (39%) of students were attending schools in the West. Full descriptive statistics are presented in Table 1.

**Table 1:** Descriptive statistics of the analytic sample.

**Variable**	**Mean (SD) or n (%)**
Student BMI (kg/m^2^)	24.9 (5.8)
Overweight (BMI ≥25) prevalence	93,025 (23.0)
Obesity (BMI ≥30) prevalence	58,183 (14.4)
Gender identification (%)	
Female	272,516 (67.3)
Male	125,146 (30.9)
Non-binary	7,325 (1.8)
Age (years)	22.5 (6.0)
Race/ethnicity (%)	
White	248,290 (61.3)
Black	18,813 (4.6)
Hispanic/Latino	53,391 (13.2)
Asian/Pacific Islander	55,886 (13.8)
Other	28,607 (7.1)
Hours worked per week (%)	
0 hours	160,863 (39.7)
1–9 hours	68,723 (17.0)
10–19 hours	77,089 (19.0)
20–29 hours	50,967 (12.6)
30–39 hours	17,487 (4.3)
40 hours	16,773 (4.1)
More than 40 hours	13,085 (3.2)
Two-year school students (%)	19,635 (4.8)
Public school students (%)	267,799 (66.1)
MSI students (%)	76,094 (18.8)
Students by US Region (%)	
Northeast	79,151 (19.5)
Midwest	77,565 (19.2)
South	89,250 (22.0)
West	159,021 (39.3)
Survey Wave	
Fall 2015	19,022 (4.7)
Spring 2016	91,100 (22.5)
Fall 2016	31,615 (7.8)
Spring 2017	60,580 (15.0)
Fall 2017	30,239 (7.5)
Spring 2018	84,849 (21.0)
Fall 2018	24,140 (6.0)
Spring 2019	63,442 (15.7)

*Note:* Unit of observation is students (n = 404,987) from 445 unique schools; two-year schools n = 34, public schools n = 266, MSI n = 68

Linear mixed effects model results are presented in Table 2 (raw estimates). Main effects for BMI of students attending two-year schools (vs. four-year schools), public schools (vs. private schools), and schools in the Midwest (vs. Northeast) were significant and positive, indicating greater BMIs among these subgroups in comparison to the reference. Second, no difference was found for BMI between MSI and non-MSI students, except for in the South where the mean BMI was higher for MSI students. Of clinical significance, the confidence intervals for all school type/region combinations included BMI > 25.0 (overweight).

**Table 2:** Linear mixed model regression results (raw estimates) predicting student BMI.

**Student BMI**	**β (CI)**	**P-value**
Two-year schools	0.052 (0.003, 0.102)	0.04*
Public Schools	0.032 (0.015, 0.050)	<0.001***
MSI	−0.010 (−0.022, 0.003)	0.15
Region		
Northeast (ref.)	--	--
Midwest	0.017 (0.001, 0.322)	0.04*
South	0.009 (−0.007, 0.025)	0.27
West	−0.016 (−0.032, 0.001)	0.07
Two-year*Midwest	−0.025 (−0.093, 0.042)	0.47
Two-year*South	−0.020 (−0.095, 0.05)	0.61
Two-year*West	−0.040 (−0.093, −0.012)	0.14
Public*Midwest	−0.004 (−0.028, 0.020)	0.75
Public*South	−0.008 (−0.032, 0.015)	0.49
Public*West	−0.003 (−0.027, 0.021)	0.82
MSI*Midwest	0.007 (−0.050, 0.065)	0.80
MSI*South	0.024 (0.003, 0.045)	0.03*
MSI*West	0.008 (−0.007, 0.023)	0.31

**Covariates**		

Age	0.006 (0.006, 0.006)	<0.001***
Gender		
Female (ref.)	--	--
Male	0.021 (0.019, 0.022)	<0.001***
Non-binary	0.040 (0.035, 0.044)	<0.001***
Race/ethnicity		
White (ref.)	--	--
Black	0.067 (0.064, 0.070)	<0.001***
Hispanic/Latino	0.047 (0.045, 0.049)	<0.001***
Asian/Pacific Isl.	−0.041 (−0.043, −0.039)	<0.001***
Other	0.018 (0.015, 0.020)	<0.001***
Hours worked/week		
0 hours (ref.)	--	--
1–9 hours	−0.003 (−0.004, −0.001)	0.003**
10–19 hours	0.008 (0.006, 0.009)	<0.001***
20–29 hours	0.015 (0.013, 0.017)	<0.001***
30–39 hours	0.027 (0.024, 0.030)	<0.001***
40 hours	0.033 (0.030, 0.036)	<0.001***
>40 hours	0.038 (0.034, 0.041)	<0.001***

*Note:* Model intercept = 3.034; significance level *0.05, **0.01, ***0.001

**Fig. 1:**
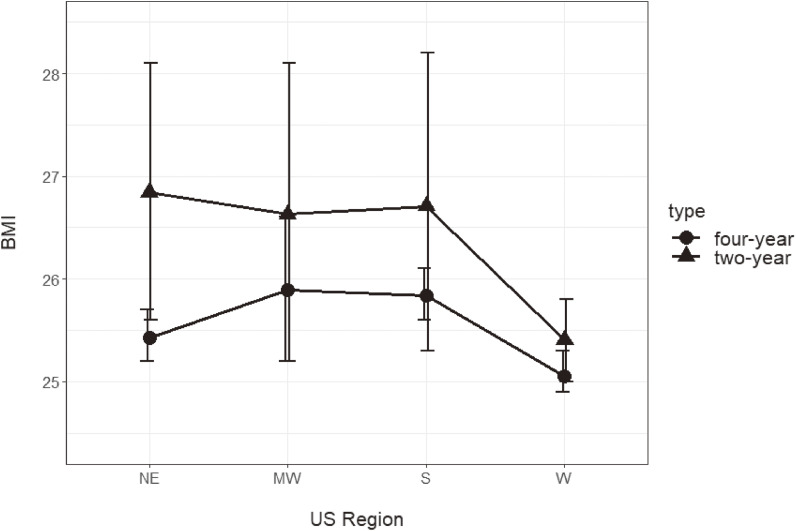
Mean BMI of two-year versus four-year school students by US region. *Note:* Two-year school students self-report higher BMIs compared to four-year school students in all regions (p = 0.04). NE two-year n = 855 (overweight/obesity prevalence = 56.6%); NE four-year n = 78,296 (overweight/obesity prevalence = 32.7%); MW two-year n = 1,049 (overweight/obesity prevalence = 56.3%); MW four-year n = 76,516 (overweight/obesity prevalence = 38.5%); S two-year n = 769 (overweight/obesity prevalence = 28.5%); S four-year n = 88,481 (overweight/obesity prevalence = 38.2%); W two-year n = 16,962 (overweight/obesity prevalence = 45.9%); W four-year n = 142,059 (overweight/obesity prevalence = 37.5%)

Across all regions, self-reported BMI among two-year school students (NE: 26.8 [95% CI: 25.6, 28.1]; MW: 26.6 [25.2, 28.1]; S: 26.7 [25.3, 28.2]; W: 25.4 [25.0, 25.8]) was higher compared to four-year school students (NE: 25.4 [25.2, 25.7]; MW: 25.9 [25.2 26.6]; S: 25.8 [25.6, 26.1]; W: 25.1 [24.9, 25.3]). See Fig. 1.

**Fig. 2:**
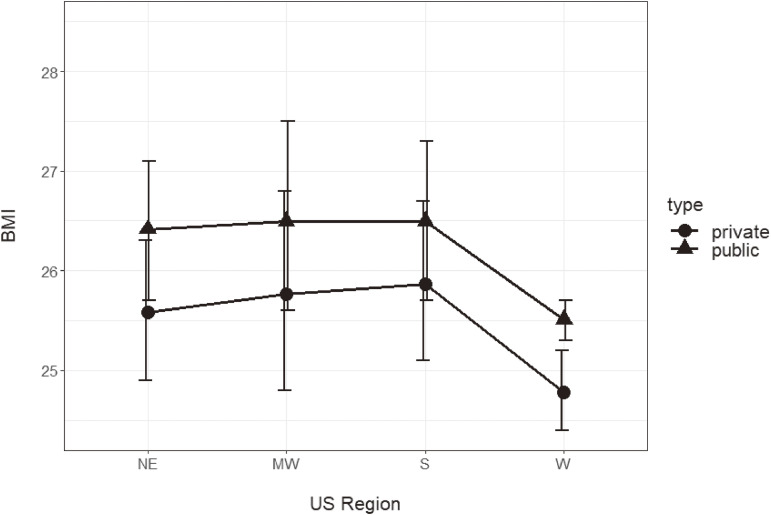
Mean BMI of public versus private school students by US region. *Note:* Public school students self-report higher BMIs compared to private school students in all regions (p < 0.001). NE public n = 25,597 (overweight/obesity prevalence = 39.4%); NE private n = 53,554 (overweight/obesity prevalence = 29.9%); MW public n = 46,747 (overweight/obesity prevalence = 42.0%); MW private n = 30,818 (overweight/obesity prevalence = 33.9%); S public n = 65,741 (overweight/obesity prevalence = 40.5%); S private n = 23,509 (overweight/obesity prevalence = 31.5%); W public n = 129,714 (overweight/obesity prevalence = 40.3%); W private n = 29,307 (overweight/obesity prevalence = 30.2%)

Across all regions, self-reported BMI among public school students (NE: 26.4 [25.7, 27.1], MW: 26.5 [25.6, 27.5], S: 26.5 [25.7, 27.3], W: 25.5 [25.3, 25.7]) was higher compared to private school students (NE: 25.6 [24.9, 26.3], MW: 25.8 [24.8, 26.8], S: 25.9 [25.1, 26.7], W: 24.8 [24.4, 25.2]). See Fig. 2.

**Fig. 3:**
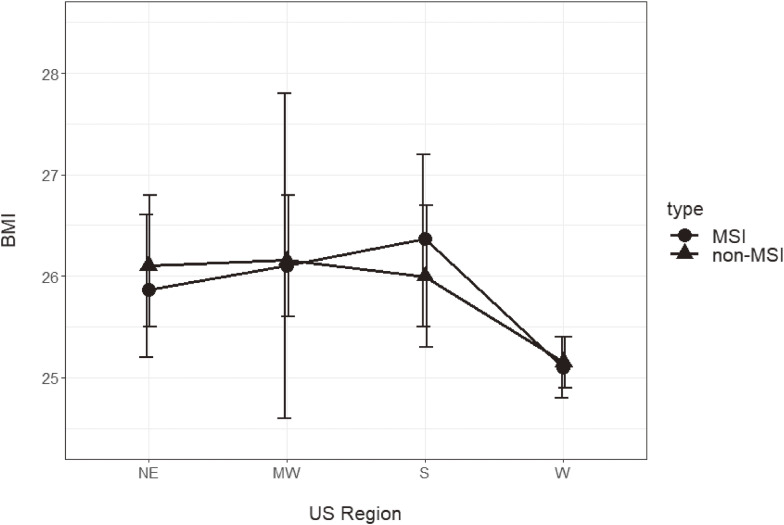
Mean BMI of MSI versus non-MSI students by US region. *Note:* Results were mixed for MSI vs. non-MSI students (p = 0.15). NE MSI n = 2,212 (overweight/obesity prevalence = 45.2%); NE non-MSI n = 76,939 (overweight/obesity prevalence = 32.6%); MW MSI n = 956 (overweight/obesity prevalence = 45.7%); MW non-MSI n = 76,609 (overweight/obesity prevalence = 38.7%); S MSI n = 5,011 (overweight/obesity prevalence = 51.0%); S non-MSI n = 84,239 (overweight/obesity prevalence = 37.3%); W MSI n = 67,915 (overweight/obesity prevalence = 42.2%); W non-MSI n = 91,106 (overweight/obesity prevalence = 35.6%)

Among MSI students, BMI of those attending MSI in the Northeast was estimated to be 25.9 (25.2, 26.6), in the Midwest: 26.1 (24.6, 27.8), in the South: 26.4 (25.5, 27.2), and in the West: 25.1 (24.8, 25.4). In comparison, BMI of students attending non-MSIs was estimated to be 26.1 (25.5, 26.8) in the Northeast, 26.2 (25.6, 26.8) in the Midwest, 26.0 (25.3, 26.7) in the South, and 25.2 (24.9, 25.4) in the West. See Fig. 3.

## Discussion

This study examined the association between college/university campus-level characteristics and student BMI controlling for age, sex, race/ethnicity, and hours worked per week, factors known to vary by school type. In support of our first and second hypotheses, we found that, students attending two-year and public schools reported higher BMIs compared to students attending four-year and private schools. In partial support of our third hypothesis, in the South only, MSI students had significantly higher BMIs compared to non-MSI students. In tentative support of hypothesis four, students attending schools in the Midwest had higher BMIs compared to students attending schools in the Northeast. Furthermore, there was a trend across school types for student BMIs in the West to be lower than in other regions. It is important to note that across all school types and regions, adjusted 95% confidence intervals for mean BMI included values greater than 25.0, the cutoff value for overweight. These results indicate that overweight BMIs are within the range of plausible BMI values for the average student, regardless of school type or region.

Previous studies indicate that college students are a population at risk of weight gain. Undergraduates gain roughly 1.55 kg, or almost 4 pounds, during their first year of college or university [6, 23], and student weight gain during a four-year degree is estimated to be between 1.6–3.0 kg, or between 3.5–6.5 pounds [6, 7]. These changes include significant increases in adiposity and reductions in lean mass [6, 7, 24], indicating a growth trajectory that is neither healthy nor sustainable. In further support of this conclusion, a growing body of evidence demonstrates that most college students do not meet dietary and physical activity guidelines [8, 25]. This may be attributed, in part, to the environments where students live, work, and play [9]. Though there is still limited understanding of how weight-related behaviors and environments differ across campus types [9], our study presents the first analyses to show that college/university student BMIs differ between public/private and two-year/four-year postsecondary institutions across the US. Although differences in BMI estimates across school types and regions in our sample were not large (∼0.5–1.5 kg/m^2^), such differences are clinically meaningful. For example, a one-unit increase in BMI is associated with increases in systolic blood pressure and decreases in HDL values [26], both of which are known risk factors for cardiovascular disease in young adults [27].

Our findings provide continued evidence that college students are in need of healthy weight loss and maintenance programming, particularly those attending two-year schools, public schools, or MSI in the South. However, colleges and universities face substantial barriers to providing such services, including resource constraints, coordination across departments, and competing student services programming (e.g., drug use, victim services, sexual health) [28]. Programs that encourage campuses to make campus wide changes that promote healthful student diet and physical activity behaviors (e.g., Partnership for a Healthier American’s Healthier Campus Initiative [29]) may be one way to target at-risk student subpopulations at the campus level. Technology-based programs that can reach many students using tailored messaging and theory-based designs (e.g., [30, 31]) are also a promising method for promoting college/university weight maintenance and healthy weight-related behaviors.

Understanding the types and locations of academic institutions most likely to serve students with elevated BMIs can provide insights for campus leaders, policy makers, and college health researchers. A better understanding of weight-related trends across campus types can ensure that students receive the services they need to succeed in maintaining a healthy weight, and an overall healthy lifestyle, during an important developmental period. Our study provides evidence that two-year, public school, and MSI (in the South only) students report higher BMIs compared to their four-year, private school, and non-MSI (in the South) counterparts, and that average students plausibly have overweight, regardless of school type or region. Future research can build on this work by surveilling student weight status across school types and US regions using nationally representative samples. In particular, more granular efforts can identify at risk student subpopulations by campus type at the state level. Such efforts are needed to comprehensively identify students in need of weight maintenance programming, as well as schools in need of funding and resources to provide such programmatic support to their student body.

## Strengths & limitations

This study offers several strengths. To the authors’ knowledge, this is the first study to test associations between campus type and student BMI using a sample of schools across all four US regions. The analytic sample was a large, diverse sample of 404,987 students from 445 schools. Furthermore, the sample included understudied and priority public health populations (i.e., two-year school students, MSI students).

Study limitations include the cross-sectional nature of the data; we cannot conclude whether students’ weight or BMIs increased while attending college or earlier in life before matriculating to college. We also do not know what specific factors may have impacted their weight during college. Future work could include student class year as a “dose-response” test of time spent in school with BMI. Although this study cannot delineate causal factors, results do indicate student subpopulations with elevated BMIs and thus most in need of weight-related programming. Second, although the ACHA-NCHA survey is disseminated to institutions across all 50 states, the dataset is not designed to be nationally representative. Participating institutions volunteer to survey their students and may be different from schools that do not participate. Finally, BMI was self-reported by students and campus-level characteristics were self-reported by campus administrators.

## Conclusion

School type/region combinations can be used as indicators of at-risk student subgroups. This cross-sectional analysis identified two-year, public school, and MSI (in the South only) students as having elevated BMIs compared to their four-year, private school, and non-MSI (in the South) counterparts, holding constant student age, gender, race/ethnicity, and hours worked per week. Nationally representative and longitudinal data are needed to further investigate these preliminary findings and to further demonstrate need for campus weight maintenance programming for students at greatest risk of overweight/obesity.

## Declarations

### Availability of data and material

The data and materials used during the current study are available from the corresponding author on reasonable request.
